# Comprehensive analysis of the FOXA1-related ceRNA network and identification of the MAGI2-AS3/DUSP2 axis as a prognostic biomarker in prostate cancer

**DOI:** 10.3389/fonc.2023.1048521

**Published:** 2023-03-14

**Authors:** Guo Yang, Xiong Chen, Zhen Quan, Miao Liu, Yuan Guo, Yangbin Tang, Lang Peng, Leilei Wang, Yingying Wu, Xiaohou Wu, Jiayu Liu, Yongbo Zheng

**Affiliations:** ^1^ Department of Urology, The First Affiliated Hospital of Chongqing Medical University, Chongqing, China; ^2^ Department of Urology, The Ninth People’s Hospital of Chongqing, Chongqing, China; ^3^ Key Laboratory of Laboratory Medical Diagnostics, Ministry of Education, Chongqing Medical University, Chongqing, China

**Keywords:** MAGI2-AS3, DUSP2, ceRNA, prostate cancer, FOXA1

## Abstract

**Background:**

Prostate cancer (PCa) is the second most common cause of cancer-related deaths in American men. Even though increasing evidence has disclosed the competitive endogenous RNA (ceRNA) regulatory networks among cancers, the complexity and behavior characteristics of the ceRNA network in PCa remain unclear. Our study aimed to investigate the forkhead box A1 (FOXA1)-related ceRNA regulatory network and ascertain potential prognostic markers associated with PCa.

**Methods:**

RNA sequence profiles downloaded from The Cancer Genome Atlas (TCGA) were analyzed to recognize differentially expressed genes (DEGs) derived from tumor and non-tumor adjacent samples as well as FOXA1^low^ and FOXA1^high^ tumor samples. The enrichment analysis was conducted for the dysregulated mRNAs. The network for the differentially expressed long non-coding RNA (lncRNA)-associated ceRNAs was then established. Survival analysis and univariate Cox regression analysis were executed to determine independent prognostic RNAs associated with PCa. The correlation between DUSP2 and immune cell infiltration level was analyzed. Tissue and blood samples were collected to verify our network. Molecular experiments were performed to explore whether DUSP2 is involved in the development of PCa.

**Results:**

A ceRNA network related to FOXA1 was constructed and comprised 18 lncRNAs, 5 miRNAs, and 44 mRNAs. The MAGI2-AS3~has-mir-106a/has-mir-204~DUSP2 ceRNA regulatory network relevant to the prognosis of PCa was obtained by analysis. We markedly distinguished the MAGI2-AS3/DUSP2 axis in the ceRNA. It will most likely become a clinical prognostic model and impact the changes in the tumor immune microenvironment of PCa. The abnormal MAGI2-AS3 expression level from the patients’ blood manifested that it would be a novel potential diagnostic biomarker for PCa. Moreover, down-expressed DUSP2 suppressed the proliferation and migration of PCa cells.

**Conclusions:**

Our findings provide pivotal clues to understanding the role of the FOXA1-concerned ceRNA network in PCa. Simultaneously, this MAGI2-AS3/DUSP2 axis might be a new significant prognostic factor associated with the diagnosis and prognosis of PCa.

## 1 Introduction

Prostate cancer (PCa) is the most prevalent cancer among men and is the second leading cause of cancer-related death, with an estimated 248,530 new cases and 34,130 deaths in 2021 in the United States (1). In recent years, despite the growing numbers of PCa patients identified through prostate-specific antigen (PSA) screening, imaging technique, and histopathological scores, approximately 25% of PCa patients will experience recurrence and metastasis, and the PCa will develop into castration-resistant prostate cancer (CRPC), leading to poor progression-free survival (PFS) (2). Therefore, it is momentous to explore effective prognostic biomarkers and/or therapeutic targets for PCa to improve our cognition in the diagnosis, prevention, and treatment of this cancer.

The Forkhead box (Fox) family, an evolutionarily conserved family of transcription factors binding to condensed, inactive chromatin and initiating chromatin remodeling, plays an essential role in human health and disease (3). The forkhead box A1 (FOXA1) protein is a member of a group of special transcription factors called pioneer factors; it is a crucial transcription factor in the initiation and development of breast, prostate, and lung cancers (4–6). In PCa, FOXA1 plays an indispensable role in androgen receptor (AR)-mediated gene regulation by interacting directly with AR and co-occupying chromatin (7). In breast cancer, silencing of FOXA1 expression by Twist1 is the main cause of Twist1-induced migration, invasion, and metastasis (8). However, previous studies of the transcriptional network for FOXA1 were mostly focused on protein-coding genes and its regulatory network of long non-coding RNAs (lncRNAs), and their role in FOXA1 oncogenic activity remains unknown.

lncRNAs refer to non-protein-coding RNAs consisting of longer than 200 nucleotides. In recent years, numerous studies have expounded that lncRNA accounts for a large proportion of microRNA in the cell (mainly in the cytoplasm) and can buffer or reduce the miRNA’s ability to degrade target gene mRNA and interfere with the translation process like a “sponge”, which is elaborated as a ceRNA network (9). There have been studies illustrating that lncRNAs play a remarkable role in a wide range of biological processes, including autophagy, infarction, cell senescence, apoptosis, cancer cell metastasis, and resistance to chemotherapeutic agents (10, 11). Other than that, they can also accommodate gene expression by a diversity of mechanisms, such as epigenetic modification, selective splicing, nuclear import, precursors to small RNAs, and even as regulators of mRNA modifiers or decoy elements (12). A growing number of aberrantly expressed lncRNAs are found in cancer, and it has shown promise as a biomarker to improve early tumor detection, monitoring of tumor treatment and relapse, and so on (13, 14). Despite the lncRNA expression specificity opening up great opportunities for exploring new biomarkers and drug targets, it remains challenging to affirm lncRNA involved in regulatory networks.

MicroRNAs (miRNAs), a kind of small non-coding long single-stranded RNA with 19–25 nucleotides, can induce RNA silencing, are involved in post-transcriptional regulation of gene expression, and play an important role in a variety of cellular functions by binding to the 3’ untranslated region (UTR) of its target mRNA (15). We have learned that miRNAs mediated approximately 30% of genes in the human genome, whereas miRNA regulates post-transcriptional regulation and requires multiple RNA-binding proteins, which are beneficial to the function of miRNA in tumorigenesis (16). In 2011, Salmena et al. first put forward the competitive endogenous RNA (ceRNA) hypothesis that lncRNA mainly regulates mRNA through a ceRNA regulation mechanism, described as a new mechanism of interaction among RNAs (17). Meanwhile, increasing studies elaborated that miRNA acts as ceRNA in lncRNA–miRNA–mRNA form and is involved in various kinds of tumorigenesis, such as stomach cancer (18), breast cancer (19), kidney cancer (20), and PCa (21).

**Figure 1:**
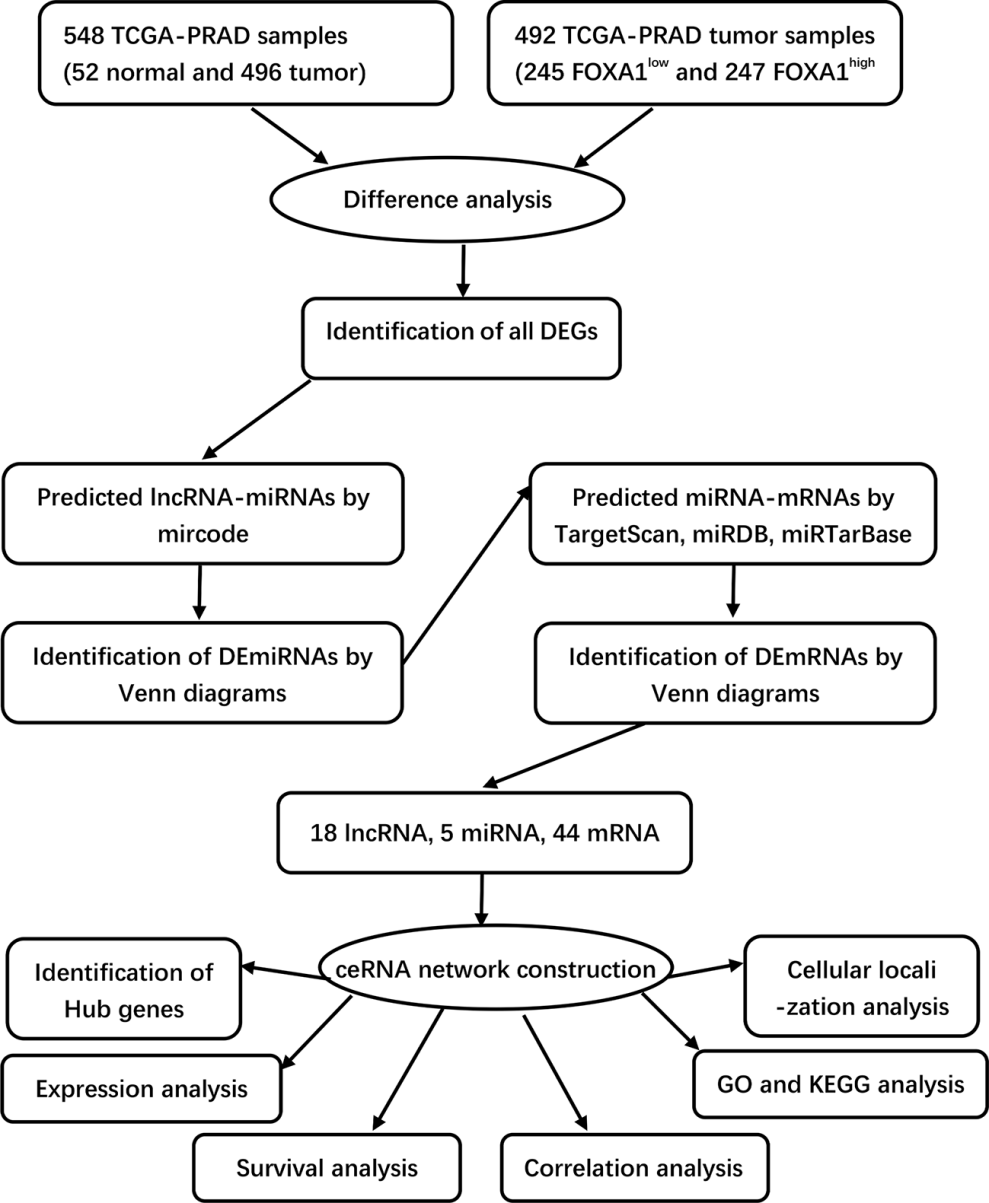
Flowchart of construction and analysis of ceRNA.

In this study, we conducted a systemic analysis of FOXA1-regulated oncogenic lncRNAs and constructed a ceRNA network related to FOXA1, as well as related to the prognosis of PCa (**Figure 1**). We chose a FOXA1-inducible lncRNA MAGI2-AS3 that is expressed in PCa and selected the FOXA1/MAGI2-AS3/DUSP2 axis in the ceRNA network as a potential prognostic model. Mechanistically, our study illuminated that the downregulated MAGI2-AS3 may function as a competing endogenous RNA for has-mir-106a or has-mir-204 to regulate the expression of DUSP2. We assessed this ceRNA axis’ value in the diagnosis of PCa and evaluated the potential relationship between DUSP2 and tumor-infiltrated immune cell levels. Furthermore, we verified the mRNA and protein levels of DUSP2 in clinical samples, and functional experiments showed that down-expressed DUSP2 inhibited the proliferation and migration of PCa cells.

## 2 Materials and methods

### 2.1 Patient samples

A total of 20 benign prostatic hyperplasia (BPH) and 20 PCa patients’ tissue specimens and corresponding anti-coagulant blood (3–4 ml) specimens were obtained from patients in the Department of Urology, the First Affiliated Hospital of Chongqing Medical University (China). Two clinicians finished the clinical diagnosis according to prostate puncture biopsy or/and PSA level. The patients provided their written informed consent to participate in this study. The studies involving human participants were reviewed and approved by the Ethics Committee of Chongqing Medical University (Ethics Approval No. 2022-K275).

### 2.2 Data preparation and processing

PCa datasets including RNA-seq and miRNA-seq from The Cancer Genome Atlas (TCGA)–PRAD datasets (https://portal.gdc.cancer.gov/) were downloaded; 52 normal and 496 tumor samples were included. Next, the data were constructed into a matrix. Ensemble names of genes were changed into symbol names using Microsoft R software (version 4.0.3) and then Homo sapiens. The GRCh38 database was used to distinguish gene types. All raw RNA-seq data (lncRNAs, miRNAs, and mRNAs) were normalized to fragments per kilobase of exon model per million mapped fragments (FPKM). The corresponding clinical data were also downloaded from the TCGA dataset and the needed information on tumor samples was extracted from it using R software.

Furthermore, we also downloaded two gene microarray datasets from Gene Expression Omnibus (GEO, http://www.ncbi.nlm.nih.gov/geo/), as they analyzed the gene expression profiles in tumor and normal tissues from PCa patients [GSE21036: normal, *n* = 28; tumor, *n* = 113 in which the Vcap cell line sample was deleted; GSE60329: normal, *n* = 28; tumor, *n* = 108 (22)]; the series matrix files were extracted from the GEO database, and the probe IDs were switched to gene symbols through corresponding platforms (GSE21036: GPL8227; GSE60329: GPL14550) to further validate our results. HPA (http://www.proteinatlas.org/) search was conducted to confirm the expression of FOXA1 in PCa at the protein level. We obtained the mutation status of FOXA1 by exploring publicly available genomic data from the cBioPortal for Cancer Genomics (http://www.cbioportal.org/).

### 2.3 Differential expression analysis

Co-expression in PCa was evaluated using TCGA. The tumor group was compared with the non-tumor group, with |log_2_(fold change FC) | > 1.0 and adjusted *p*-value < 0.05 as cutoff criteria and the “edgeR” package was used to identify differentially expressed mRNAs (DEmRNAs), differentially expressed miRNAs (DEmiRNAs), and differentially expressed lncRNAs (DElncRNAs). Because there is slightly less DEGs between the group of FOXA1^high^ and FOXA1^low^, when performing differential expression analysis in the two groups in PCa samples, we detected DElncRNAs with a threshold of |logFC| > 0.5 and *p* < 0.05, DEmiRNAs with a threshold of |logFC| > 0.3 and *p* < 0.05, and DEmRNAs with a threshold of |logFC| >0.5 as the cutoff criteria and *p* < 0.05. All *p*-values were corrected for statistical significance using the false discovery rate (FDR) with multiple testing (Benjamini–Hochberg method). The FDR significance level was 0.05. Volcano plots of DERNAs, including DElncRNAs, DEmiRNAs, and DEmRNAs, were visualized using the “ggplot2” software package. Heatmap clusters were drawn using TBtools software (version 1.051).

### 2.4 LncRNA–miRNA–mRNA network construction

Based on the hypothesis that lncRNAs can indirectly regulate mRNA expression by competing with miRNAs as natural sponges in the cytoplasm, a ceRNA network was built through the following steps: (1) the Venn Diagram package in R software was developed to identify all DEGs; (2) miRcode (http://mircode.org/) was utilized to forecast the potential miRNAs targeted by DElncRNAs and the lncRNA–miRNA interaction pairs, and a Venn Diagram was used to compare the target genes with DEmiRNAs, which overlapped with DEmiRNAs selected for the next analysis; (3) miRDB (http://www.mirdb.org/), miRTarBase (http://mirtarbase.mbc.nctu.edu.tw/php/index.php), and TargetScan (http://www.targetscan.org/) were used to predict the target genes of the DEmiRNAs and construct the miRNA–mRNA interaction pairs; (4) the Venn Diagram package in R software was utilized to compare the target genes with DEmRNAs, and the target genes overlapped with DEmRNAs that were picked out for the next analysis; and (5) by integrating the lncRNA–miRNA pairs with miRNA–mRNA pairs, we constructed the lnRNA–miRNA–mRNA triple regulatory network.

The DElncRNA sequences were acquired by seeking the LNCipedia (https://lncipedia.org/) database, while the lncLocator (http://www.csbio.sjtu.edu.cn/bioinf/lncLocator/) database was utilized to predict the DElncRNA cellular localization according to the sequences. The hub triple regulatory network was identified by the Cytoscape plug-in cytoHubba tool. The generated networks were visualized by Cytoscape software (version 3.7.0, https://www.cytoscape.org/).

### 2.5 Functional enrichment analysis

Gene Ontology (GO) is a database constructed by the Association for Gene Ontology. GO annotations can be divided into three categories, comprising biological process (BP), cellular components (CC), and molecular function (MF). Kyoto Encyclopedia of Genes and Genomes (KEGG) is a comprehensive database integrating genomic, chemical, and systemic functional information, of which the KEGG pathway is specifically dedicated to storing genetic pathway information between different species. To explore the biological functions of the candidate gene modules, the R software package “Cluster Profiler” (version 3.14.3) was used to conduct analysis for these genes. The minimum gene set was set to 5 and the maximum gene set was set to 500. *p* < 0.05 and FDR < 0.25 were considered statistically significant. Moreover, the GEO dataset (GSE60329) was utilized to analyze the DUSP2 of tumor immune-related pathways *via* gene set enrichment analysis (GSEA).

### 2.6 Survival analysis and construction of a specific prognosis model for PCa

We used Sangerbox 3.0 (http://vip.sangerbox.com) to perform Kaplan–Meier analysis on DElncRNAs, DEmiRNAs, and DEmRNAs in the ceRNA network to determine the relationship between the expression and the PFS of PCa patients in the TCGA database. Log-rank test was used to assess statistical significance and *p* < 0.05 was considered statistically significant. Additionally, univariate Cox regression analysis was utilized to analyze the association between candidate genes in the ceRNA network and clinicopathological features of PCa patients.

### 2.7 Immune infiltrate levels and immunotherapy analysis of DUSP2

An online tool—tumor immune estimation resource (TIMER) (https://cistrome.shinyapps.io/timer/)—was applied to visualize the correlation between the expression of DUSP2 and the infiltrating level of different subsets of immune cells. Moreover, immunotherapy and drug sensitivity analysis was exerted between low DUSP2 expression and high DUSP2 expression groups *via* TICA (https://tcia.at/home) and pRRophetic R package, respectively.

### 2.8 Reverse transcription and quantitative real-time PCR

Total RNA was extracted from tissue and blood samples using TRIzol (Takara), and reverse transcription was performed by the Prime Script RT reagent kit according to the manufacturer’s protocols (Takara). Real-time PCR was performed with the SYBR Premix Ex Taq™ II kit (Takara). The expression of genes was calculated by the comparative 2^−ΔΔCT^ method. The sequences of the primers were as follows: MAGI2-AS3 sense, 5′-GAGCACA TATCAATGAAGAA-3′ and antisense, 5′-ATCACCATCTCTCAAC TC-3′; and β-actin sense, 5′-TGACGT GGACATCCGCAA AG-3′ and antisense, 5′-CTGGA AGGTGGACAGCGAGG-3′. All gene expressions were normalized against β-actin and experiments were performed in triplicate at least.

### 2.9 Immunohistochemistry

All tissue samples were fixed in 10% neutral formalin, embedded in paraffin, and cut into 5-μm-thick sections. The tissue slides were prepared and deparaffinized by baking in an oven at 60°C for 1 h. The slices were dewaxed in xylene, rehydrated in a graded series of alcohols, and then antigen unmasking was done in a boiling container with sodium citrate buffer for 20 min, blocked with goat serum. Slides were then stained with DUSP2 antibody (1:500, Sigma, SAB4300841) overnight at 4°C. After PBS washing, the slices were incubated with goat anti-rabbit secondary antibodies for 1 h at room temperature. After adding substrate and hematoxylin staining, slides were covered and observed using a microscope. ImageJ software was used to analyze the staining intensity and positive rate score. BPH and tumor tissues were categorized as high and low expression according to whether staining cells ≥5% (23).

### 2.10 Cells, cell culture, and transfection

22RV1was purchased from the Cell Bank of the Chinese Academy of Sciences (Shanghai, China). Cells were cultured in RPMI 1640 (Gibco, USA) added with 10% FBS (Gibco, Thermo Fisher Scientific) under atmosphere containing 5% CO_2_ at 37°C. The full-length cDNA of DUSP2 was synthesized and cloned into the lentiviral vector pcDNA3.1(+) to form overexpressing DUSP2 plasmid (oe-DUSP2). shRNA sequences characteristically targeting DUSP2 were cloned into the pLKO.1-GFP vector to generate the sh-DUSP2 plasmids, with sh-NC as the negative control. All plasmids were designed and synthesized by Tsingke Biotechnology (Beijing, China). The recombinant and empty vectors were packaged into lentiviral particles and transfected into PCa cells. Cell transfection by Lipofectamine 3000 was performed according to the manufacturer’s instructions (Invitrogen, Thermo Fisher Scientific).

### 2.11 Cell counting kit-8 assay

22RV1 cells transfected with shRNA or vector were grown on a 96-well plate. The cell number in each well was 1 × 10^3^ added with 100 μl of medium. After 24 h of inoculation, the CCK-8 reagent solution (10 µl; Hanbio Technology) was dropped into the plate followed by incubation for 2 h. Then, the absorbance at 450 nm of each well was detected by a microplate reader (Bio-Rad Laboratories, Inc.). The remaining plates were then sampled for these detection steps on days 2, 3, and 4 after inoculation.

### 2.12 Colony formation assay

The cells were digested with 0.25% trypsin and counted, and then the cell suspension was seeded on a six-well plate (1 × 10^3^/well). The cells were then cultured for 10–14 days. The cell colonies were fixed with 4% paraformaldehyde for 20 min and stained with 1% crystal violet. The size and number of the colonies were observed and imaged.

### 2.13 Cell migration assay

For the transwell assay, the transfected cell suspensions (2.5 × 10^5^ cells per well), which were starved for 6–8 h, were seeded in the upper chamber with 400 μl of serum-free medium to measure the cell migration ability. Six hundred microliters of 10% FBS was added to the bottom chamber. The plate was incubated for 1 day and stained with crystal violet. Cell counting was performed under a microscope (Nikon, Japan). Five fields were randomly selected for each treatment group.

### 2.14 Statistical analysis

GraphPad Prism (version 8.0, San Diego, CA, USA) and SPSS 23.0 software (SPSS, Chicago, IL, USA) were used to analyze the obtained data. Results are presented as mean ± standard deviation. The association among categorical variables was analyzed by Student’s *t*-test, chi-square test, and one-way ANOVA. Kaplan–Meier method and Pearson analysis were used to assess the statistical significance. *p* < 0.05 was considered as a statistically significant difference.

## 3 Results

### 3.1 The tumorigenesis role and prognostic value of FOXA1 overexpression in PCa

**Figure 2:**
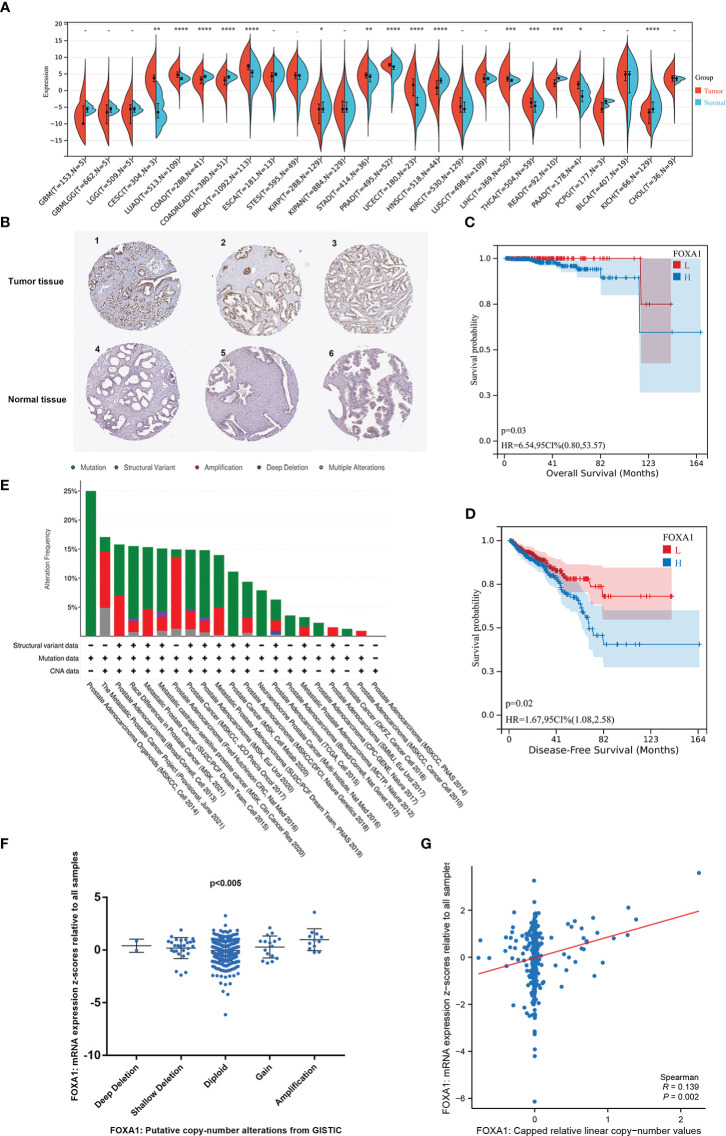
The carcinogenic role of FOXA1 in prostate cancer. **(A)** Expression distribution of FOXA1 in pan-cancer tissues. **(B)** Validation of the expression of FOXA1 on the translational level by the Human Protein Atlas database (immunohistochemistry). **(C, D)** Kaplan–Meier survival analysis of the relationship between FOXA1 expression and cancer patient prognosis in the TCGA cohort (H = FOXA1 high expression, *n* = 247; L = FOXA1 low expression, *n* = 245). **(E)** The distribution of FOXA1 genomic alterations in 21 prostate studies is shown on a cBioPortal OncoPrint plot. **(E, F)** The association between FOXA1 copy number and mRNA expression is displayed in the dot plot **(F)** and correlation plot **(G)** by cBioPortal. *P <0.05, **P < 0.01, ***P < 0.001, ****P<0.0001.

To evaluate the probable role of FOXA1 in PCa, we analyzed and discovered that FOXA1 was upregulated in PCa tissues more than normal tissues (**Figure 2A**). Similarly, the upregulated FOXA1 was also verified by immunohistochemistry (IHC) staining from the HPA database (**Figure 2B**, **Supplementary Table 1**). Since FOXA1 is aberrantly overexpressed in PCa specimens, we then analyzed the clinical significance of FOXA1 expression in PCa patients. Our data revealed that enhanced expression of FOXA1 significantly correlated with poor overall survival (OS) and disease-free survival (DFS) in the patients of the cohort (**Figures 2C, D**). These results were consistent with those of previous studies (24), revealing significantly upregulated FOXA1 in PCa tissues and showing a prognostic value.

Moreover, to investigate the possible mechanism of the high FOXA1 expression in PCa, the genomic and copy numbers of FOXA1 were analyzed. As a result of our analysis on cBioPortal, we created an OncoPrint plot exhibiting the alteration frequency of the FOXA1 gene in 21 studies of prostate datasets (**Figure 2E**). Moreover, a higher level of mRNA expression was discovered in PCa samples with FOXA1 gain or amplification than in those with diploid or deletion (**Figure 2F**). Moreover, we found a positive correlation between FOXA1 copy number value and mRNA expression in PCa samples (**Figure 2G**).

Taken together, the combined data testify that FOXA1 expression is elevated in PCa and that the gain and amplification of FOXA1 copy numbers are likely to be key factors that make a contribution to the upregulation of FOXA1 and play a crucial role in the progression of PCa.

### 3.2 Identification of differentially expressed lncRNAs, miRNAs, and mRNAs

**Figure 3:**
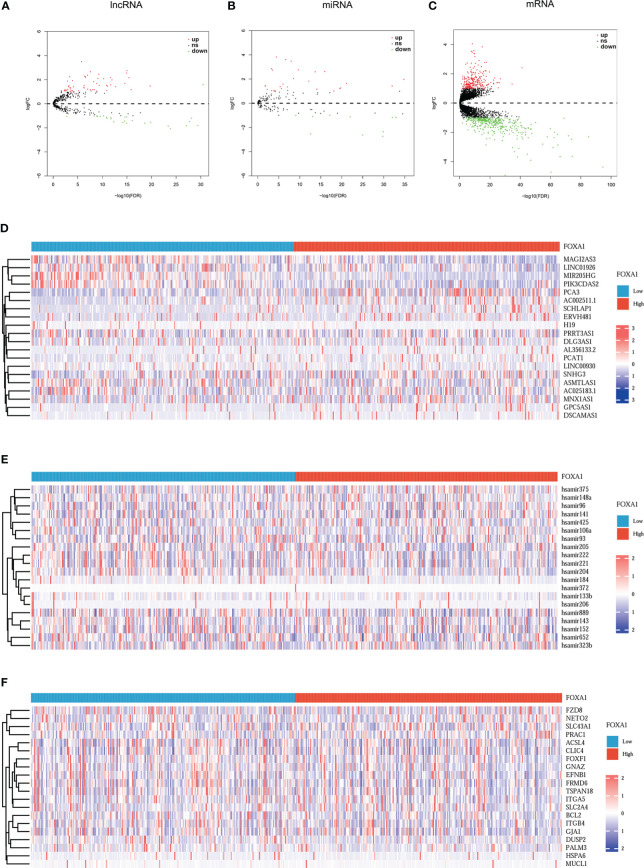
Volcano plots and heatmap plots of DElncRNAs, DEmiRNAs, and DEmRNAs between the expression of FOXA1^high^ and FOXA1^low^ in PCa samples. Red represents upregulated genes and blue indicates downregulated genes. **(A–C)** The volcano plots describe **(A)** 81 DElncRNAs (|log_2_fold change| > 0.5 and adjusted *p*-value < 0.05), **(B)** 38 DEmiRNAs (|log_2_fold change| > 0.3 and adjusted *p*-value < 0.05), and **(C)** 653 DEmRNAs (|log_2_fold change| > 0.5 and adjusted *p* value < 0.05). **(D–F)** The horizontal axis of the heatmap indicates the samples, and the vertical axis of the heatmap indicates 15 significant DEGs.

Based on the above analysis, the ceRNAs associated with FOXA1 can potentially serve as a prognostic indicator for patients with PCa. We should be aware that the difference between the expression levels in PCa samples with FOXA1^high^ and FOXA1^low^ expression groups and those in tumor and normal groups is opposite (25). To verify this hypothesis, first, we screened DEmiRNAs, DElncRNAs, and DEmRNAs in PCa samples between the high and low FOXA1 expression group. We sorted out 81 DElncRNAs (55 upregulated and 26 downregulated), 38 DEmiRNAs (10 upregulated and 28 downregulated), and 653 DEmRNAs (248 upregulated and 405 downregulated). Simultaneously, the tumor group was compared with the non-tumor group, and we identified a total of 178 DElncRNAs (129 upregulated and 49 downregulated), 123 DEmiRNAs (69 upregulated and 54 downregulated), and 1,059 DEmRNAs (653 upregulated and 406 downregulated). We selected the mRNAs, miRNAs, and lncRNAs that were upregulated or downregulated in the first place and mapped their expression on the volcano (**Figures 3A–C**; **Supplementary Figures 1A–C**), while the heatmaps depict the expression of 20 momentous variable genes in PCa samples with FOXA1^high^ and FOXA1^low^ expression, as well as in tumor and normal samples (**Figures 3D–F**; **Supplementary Figures 1D–F**). Second, the Venn diagram for DElncRNA, DEmRNA, and DEmiRNA between the two groups was conducted individually to obtain the common DEGs, which were used for further analysis.

### 3.3 Construction of the triple regulatory network in lncRNA–miRNA–mRNA and identification of the hub gene

**Figure 4:**
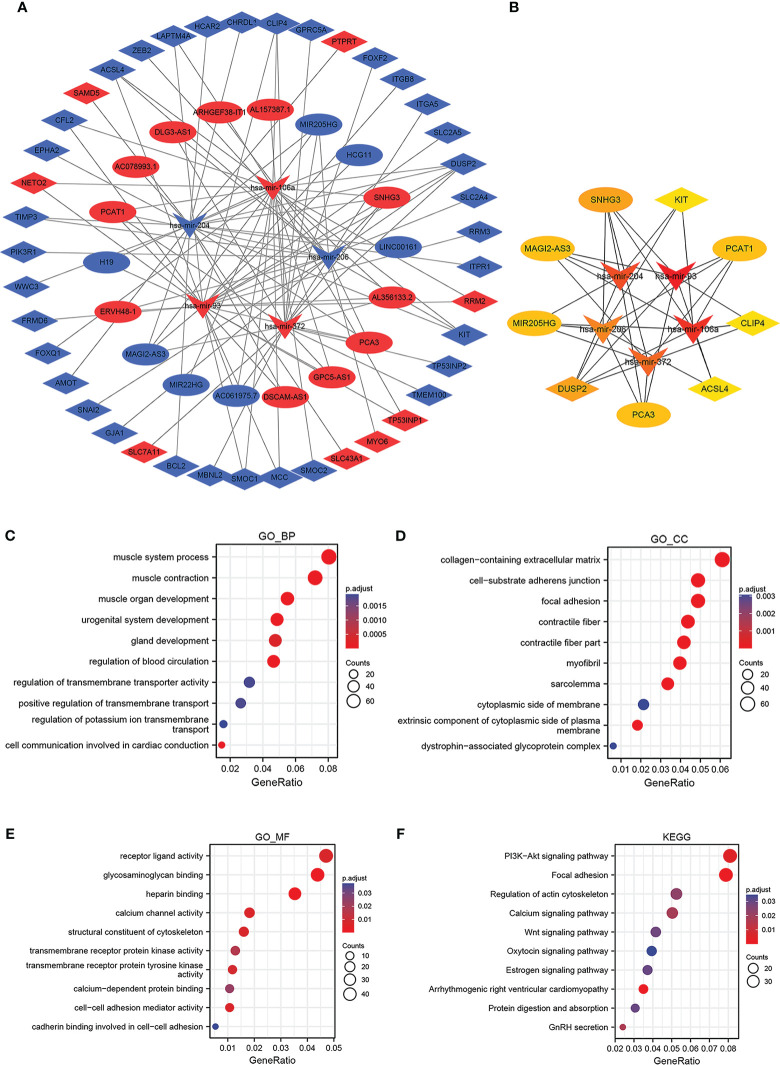
Construction and functional enrichment analysis of the lncRNA–miRNA–mRNA triple regulatory network. The ellipses denote lncRNAs, diamonds denote mRNAs, and round rectangles denote miRNAs. **(A)** The triple regulatory network in PCa. **(B)** Thirteen hub genes are in this network with a score of >2. **(C–F)** Functional enrichment analysis (GO and KEGG) of the DEmRNAs in the network.

To establish a triple regulatory network in PCa, in the first place, we identified potential miRNAs targeting lncRNAs by putting the 18 DElncRNAs into the miRcode database; after taking the intersection with the common DEmiRNAs, five of the predicted miRNAs were selected. Secondly, we identified the downstream target mRNAs regarding the five DEmiRNAs by using the databases of miRDB, miRTarBase, and TargetScan together. To enhance the validity of the predictions, we also sought to ascertain candidate mRNAs that were only shared by the three databases. The results revealed that 44 of the predicted DEmRNAs conformed after taking the intersection with the common DEmRNAs. Finally, by integrating the lncRNA–miRNA pairs with miRNA–mRNA pairs, we constructed the lnRNA–miRNA–mRNA triple regulatory network; a total of 18 lncRNAs (11 upregulated and 7 downregulated), 5 miRNAs (3 upregulated and 2 downregulated), and 44 mRNAs (8 upregulated and 36 downregulated) were included (**Figure 4A**). In detail, 5 miRNA nodes, 18 lncRNA nodes, and 44 mRNA nodes are included in the network.

The hub genes were identified in the regulatory network using cytoHubba, and the results showed that five lncRNAs (MAGI2-AS3, MIR205HG, PCAT1, PCA3, and SNHG3), five miRNAs (has-mir-106a, has-mir-204, has-mir-206, has-mir-372, and has-mir-93) and four mRNAs (DUSP2, CLIP4, KIT, and ACSL4) were identified (**Figure 4B**). These genes, which were regulated by FOXA1, could form a ceRNA network to mediate PCa progression.

### 3.4 Functional enrichment analysis of DEmRNAs

For further insight into potential functions associated with the triple regulatory network, GO enrichment analyses for DEGs based on the mRNA–miRNA–lncRNA network were conducted first. On the BP level, the DEGs were mostly enriched in muscle organ development, gland development, and urogenital system development (**Figure 4C**). On the CC level, the DEGs tended to be enriched in the collagen-containing extracellular matrix and cell-substrate adherent junction (**Figure 4D**). Additionally, the DEGs were primarily enriched in receptor–ligand activity, heparin binding, and calcium channel activity on the MF level (**Figure 4E**). Afterward, we carried out KEGG enrichment analysis, and the top 10 most remarkable pathway terms were presented. The genes were largely enriched in the PI3K-Akt signaling pathway, focal adhesion, and regulation of the actin cytoskeleton (**Figure 4F**). Details of the top 10 markedly enriched pathways of GO and KEGG derived from DEmRNAs are shown in **Supplementary Table 2**.

### 3.5 The hub gene analysis as well as the selection of a model with PCa-specific prognostic value

**Figure 5:**
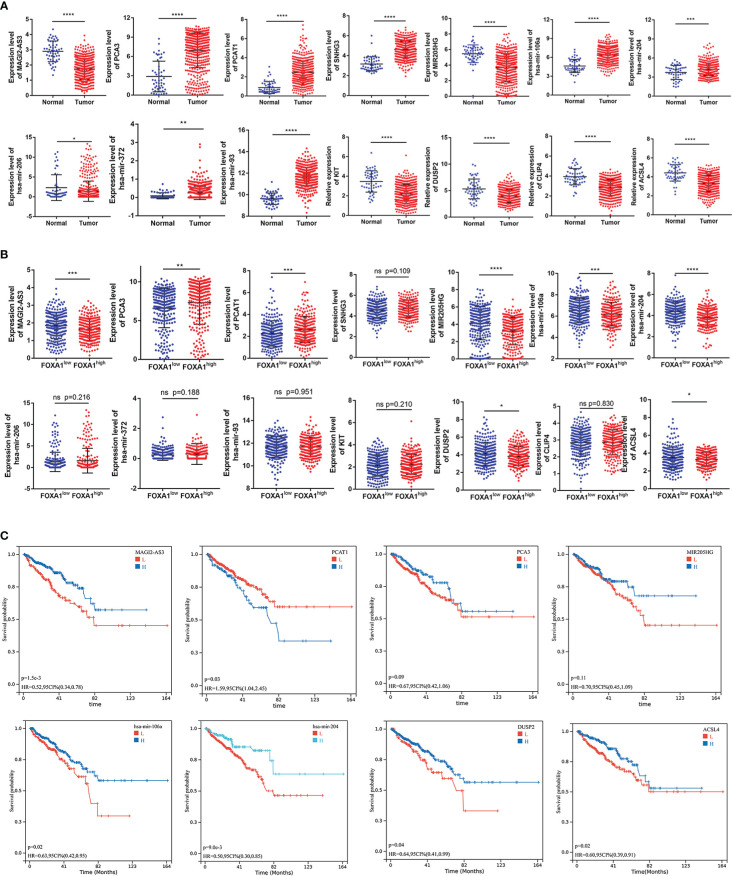
Expression and survival analysis for the hub genes. **(A)** The expression patterns of five DElncRNAs, five hub-DEmiRNAs, and four hub-DEmRNAs in PCa and adjacent normal prostate tissues and **(B)** in PCa samples with FOXA1^high^ and FOXA1^low^ expression groups. **(C)** The high- and low-expression values of hub genes were compared by a Kaplan–Meier survival curve for the TCGA prostate patient cohort. The horizontal axis indicates the overall survival time in months, and the vertical axis represents the survival rate. *P <0.05, **P < 0.01, ***P < 0.001, ****P<0.0001.

As a first step to discerning a ceRNA with great prognostic value, we investigated and visualized the relationship between the hub gene expression and clinicopathological characteristics of PCa patients, including age, TMN stage, Gleason score, and survival state (**Supplementary Figure 2A**). As studies demonstrated that transcripts within a ceRNA network are co-regulated, we first examined the expression levels across the hub triple regulatory network in PCa samples with low- and high-expression groups of FOXA1 as well as in PCa and normal prostate tissues. Our results displayed three upregulated (PCA3, PCAT1, and SNHG3) and two downregulated (MAGI2-AS3 and MIR205HG) lncRNAs, three upregulated (has-mir-106a, has-mir-372, and has-mir-93) and two downregulated (has-mir-204, and has-mir-206) miRNAs, and four downregulated (DUSP2, CLIP4, ACSL4, and KIT) mRNAs in PCa and normal prostate tissues (**Figure 5A**). In the meantime, we spotted two upregulated (MAGI2-AS3 and MIR205HG) lncRNAs, two downregulated (PCA3 and PCAT1) lncRNAs, and one undifferentiated (SNHG3) lncRNA; two downregulated (has-mir-106a and has-mir-204) and three undifferentiated (has-mir-206, has-mir-372 and has-mir-93) miRNAs; and two downregulated (DUSP2 and ACSL4) and two undifferentiated (KIT and CLIP4) mRNAs in PCa samples with FOXA1^low^ and FOXA1^high^ expression groups (**Figure 5B**).

On the other side, GSE21036 and GSE60329 were analyzed for the verification of these RNAs’ expression levels. These findings are almost consistent with the analysis that we elucidated above (**Supplementary Figures 2B–D**). Subsequently, we accomplish Kaplan–Meier analysis and a log-rank test for PFS in PCa patients to determine whether these RNAs were associated with PCa prognosis. In general, two DElncRNAs (MAGI2-AS3 and PCAT1), two DEmiRNAs (has-mir-106a and has-mir-204), and two DEmRNAs (DUSP2 and ACSL4) were identified to be associated with prognosis based on *p* < 0.05 (**Figure 5C**).

### 3.6 Construction and verification of the ceRNA network

**Figure 6:**
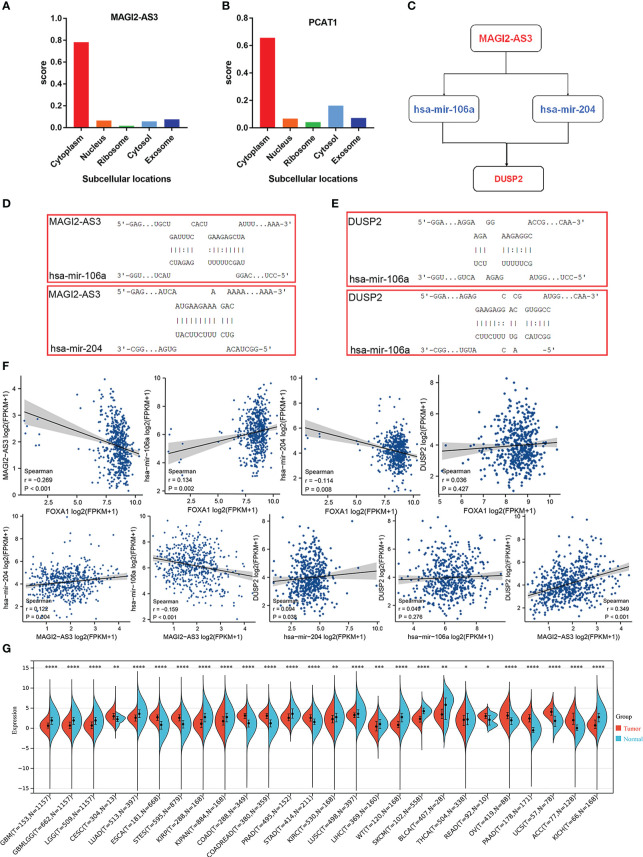
Construction and verification of the ceRNA network. **(A, B)** The cellular localization for two hub-lncRNAs (MAGI2-AS3 and PCAT1) was predicted using lncLocator. **(C)** Schematic model of ceRNA. Blue indicates upregulated; red indicates downregulated. **(D, E)** Base pairing between has-mir-106a and has-mir-204 and the target site in the MAGI2-AS3 and DUSP2 3’ UTR predicted by RNAInter, respectively. **(F)** Correlation analysis between these four predictive RNAs and FOXA1 in PCa. **(G)** Expression distribution of DUSP2 in pan-cancer tissues. *P <0.05, **P < 0.01, ***P < 0.001, ****P<0.0001.

Because cellular localization of lncRNAs determined the underlying mechanisms, we enforced the lncLocator analysis on the two DElncRNAs to predict the subcellular localization. The results showed that MAGI2-AS3 and PCAT1 are located predominantly in the cytoplasm (**Figures 6A, B**), but given the consideration that PCAT1 had been comprehensively studied in PCa, we focus on MAGI2-AS3 only. Overall, these data reminded us that MAGI2-AS3 may function as a ceRNA to enhance the expression of DUSP2 through sponging has-mir-106a/has-mir-204 (**Figure 6C**). The target sites in the MAGI2-AS3 and DUSP2 3’ UTRs were predicted to pair with has-mir-106a and has-mir-204 by RNAInter, respectively (**Figures 6D, E**; **Supplementary table 3**). Pearson correlation analysis was carried out to verify the correlation of the independent prognostic lncRNA and mRNA factors, and the correlation of these RNAs with FOXA1 expression. We found that there is a strong positive correlation between MAGI2-AS3 and DUSP2; equally, FOXA1 had a strong negative correlation with MAGI2-AS3 (**Figure 6F**). These results further confirmed the regulatory network we constructed. At present, no study has yet to disclose the relationship between MAGI2-AS3 and DUSP2, or FOXA1 and MAGI2-AS3 in cancer. Consequently, the FOXA1/MAGI2-AS3/DUSP2 axis in the ceRNA network was singled out as a potential prognostic model for the next step analysis. We then performed a simple pan-cancer analysis of DUSP2 expression, which showed that DUSP2 expression was not tissue-specific and was downregulated in some cancer tissues when compared with normal tissues, including PCa (**Figure 6G**).

### 3.7 Exploring the relationship between DUSP2 expression and immune infiltration or immunotherapy in PCa

**Figure 7:**
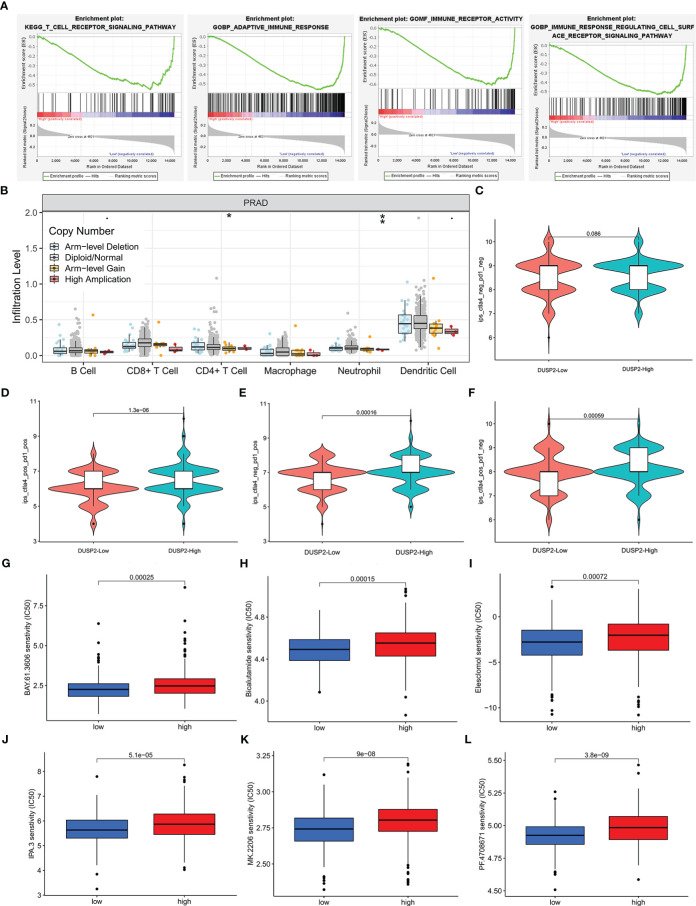
Immune infiltrate levels as well as immunotherapy or chemosensitivity analysis of DUSP2. **(A)** GSEA for DUSP2 with GSE60329. **(B)** Correlation of DUSP2 expression with immune infiltration level in PCa. **(C–F)** Low DUSP2 expression was negatively correlated with CTLA4 or/and PD1-positive levels. **(G–L)** DUSP2 acted as a potential predictor for chemosensitivity as low DUSP2 was related to a lower IC_50_ for chemotherapeutics (low = low DUSP2 expression, high = high DUSP2 expression). *P <0.05, **P < 0.01

To evaluate the potential relationship between DUSP2 expression and tumor immunity in PCa, GSEA was conducted using GSE60329, and the results showed that T-cell receptor signaling pathway, adaptive immune response, immune receptor activity, and immune response regulating cell surface receptor signaling pathway were enriched in the low DUSP2 expression group (**Figure 7A**). The following analysis was conducted by using the TIMER tool. The “SCNA” module analysis showed that CD4^+^ T cells’ and neutrophil cells’ immune infiltration levels were elevated after DUSP2 arm-level deletion (**Figure 7B**). These altogether suggested that DUSP2 was important for immune cell infiltration in tumor pathology.

Since immune checkpoint inhibitors (ICIs) have tremendous potential for treating cancer, we investigated whether the DUSP2 expression was related to ICI-related biomarkers and discovered that high expression of DUSP2 was positively correlated when both T-lymphocyte-associated protein 4 (CTLA-4) and programmed cell death protein 1 (PD-1) were positive, whereas there were no statistical differences when both PD-1 and CTLA-4 were negative (**Figures 7C–F**). Finally, we attempted to identify associations between DUSP2 and the efficacy of chemotherapeutics in the TCGA project of the PRAD dataset. Results showed that a lower DUSP2 expression was associated with a lower half-inhibitory concentration (IC_50_) of chemotherapeutics such as BAY.61.3606, Bicalutamide, Elesclomol, IPA.3, MK.2206, and pf.4708671 (**Figures 7G–L**), which indicated that the model acted as a potential predictor for chemosensitivity.

### 3.8 Validation of the lncRNA–miRNA–mRNA network in tissue and blood sample

**Figure 8:**
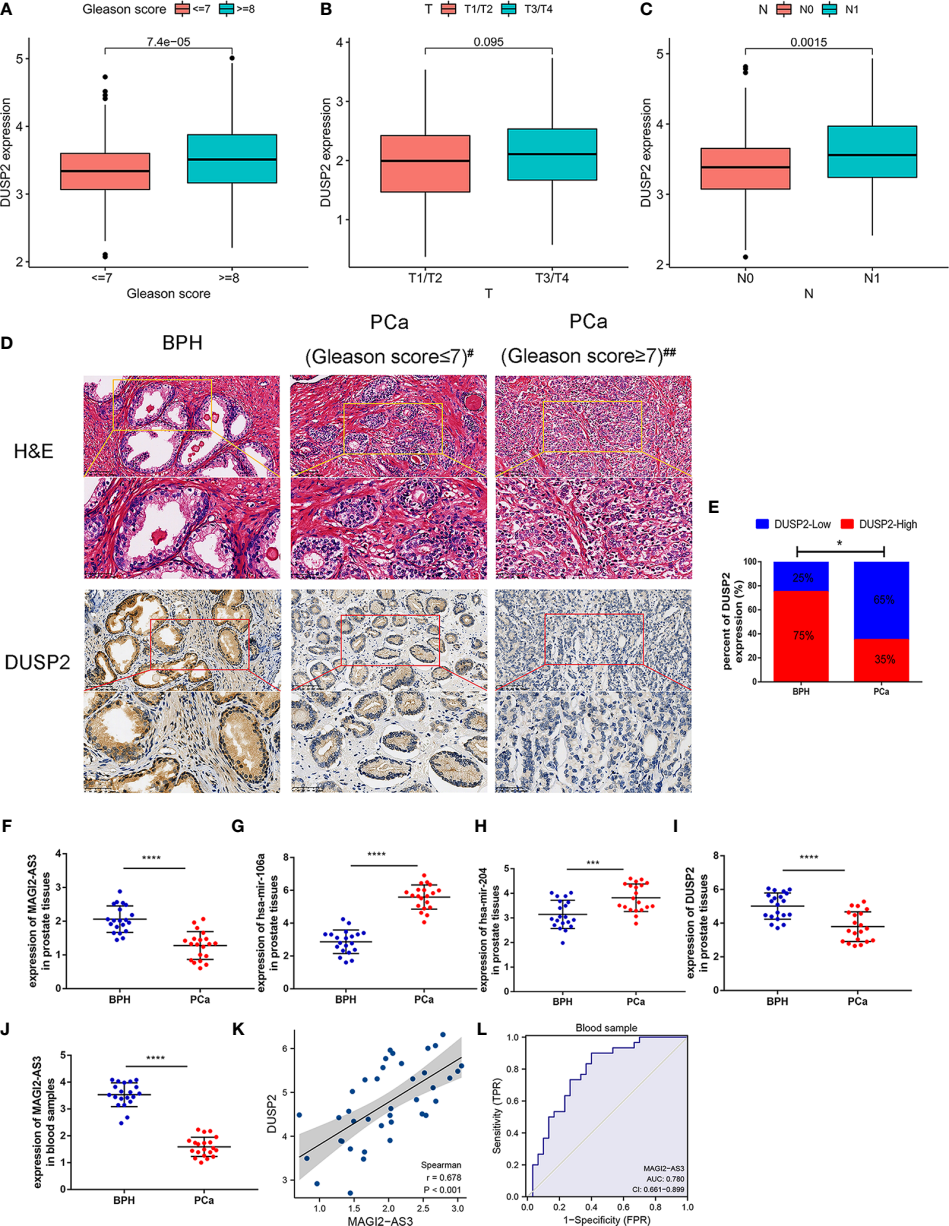
Validation of the lncRNA–miRNA–mRNA network in tissue and blood sample. **(A–E)** The correlation between DUSP2 expression and the clinical characteristics of PCa. **(F)** Representative hematoxylin and eosin (H&E) staining and immunohistochemical (IHC) staining in 20 PCa tissue samples and 20 BPH tissue samples; ^#^Gleason score = 7(3 + 4), ^##^Gleason score = 7(4 + 3). **(G)** The percentage of DUSP2 staining intensity in 20 PCa patients and 20 BPH patients. **(H–K)** The network molecular expression level in fresh BPH and PCa tissues. **(L)** Positive correlation between MAGI2-AS3 and DUSP2 in prostate tissues; *r* = 0.362. **(M)** The expression of MAGI2-AS3 in the blood of patients with PCa and BPH. **(N)** ROC curves were plotted to evaluate the predictive accuracy of MAGI2-AS3 expression for PCa diagnosis; AUC = 0.780. *p < 0.05, ***p < 0.001, ****p < 0.0001.

First, we studied the relationship between the expression of DUSP2 and the clinical characteristics of PCa. Results revealed that decreased DUSP2 expression was associated with a higher T stage, a higher Gleason score, and a higher incidence of lymph node metastases (**Figures 8A–C**, **Supplementary Table 3**). These findings inferred that the dysregulated DUSP2 participated in the progression of PCa. The expression of DUSP2 was examined by clinical samples. The statistical results presented that the proportion of DUSP2 defined as high expression in BPH tissues was observably higher than that in PCa tissues (**Figures 8D, E**), revealing that DUSP2 expression was lower in the tumor tissues than in the BPH tissues, and the expression was weakened by carcinoma progression, which was consistent with our bioinformatics analysis

To further verify the expression of the MAGI2-AS3/DUSP2 axis at the mRNA level, RNA was extracted from the tissue samples and detected by RT-PCR. The results showed increased expression of has-mir-106a and has-mir-204 in PCa patients in contrast to those with BPH, but decreased MAGI2-AS3 and DUSP2 (**Figures 8F–I**). Furthermore, we detected the relative expression of MAGI2-AS3 in blood samples to assess its role in PCa; the results showed that the MAGI2-AS3 mRNA concentrations were notably reduced in PCa patients than in BPH (**Figure 8J**). We also observed a positive correlation between DUSP2 expression and MAGI2-AS3 expression in tissue samples (**Figure 8K**). Interestingly, these results are consistent with previous analyses. The area under the curve (AUC) values were estimated based on ROC curve analysis. AUC value was 0.780 (95% CI: 0.661–0.899) (**Figure 8L**). Hence, the results manifested a good specificity and sensitivity and hinted that MAGI2-AS3 is of value in distinguishing PCa from BPH.

### 3.9 DUSP2 regulated the proliferation and migration of PCa cells

**Figure 9:**
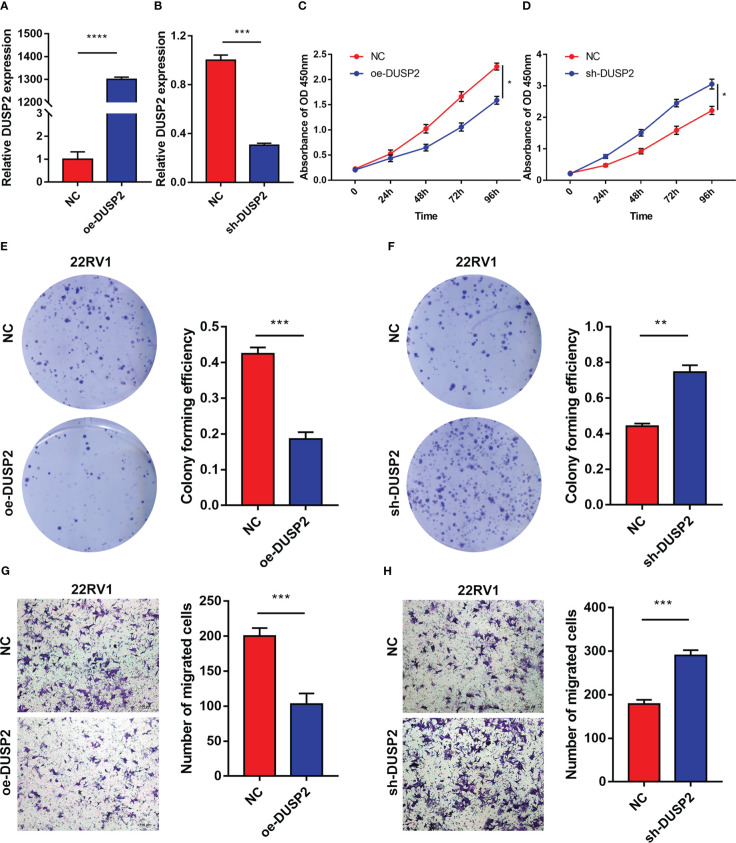
The DUSP2 regulated the proliferation and migration of PCa cells. **(A, B)** q-PCR was performed to determine the overexpression and knockdown efficiencies of DUSP2 in 22RV1 cells. **(C–F)** CCK8 assays and colony formation assays were used to determine the proliferation ability of 22RV1 cells after overexpressing or silencing DUSP2. **(G, H)** Migration capacity of cells with DUSP2 overexpression or knockdown based on transwell assays. *P <0.05, **P < 0.01, ***P < 0.001, ****P<0.0001

Since DUSP2 has not been reported in PCa, it is worthy to explore the biological behavior of DUSP2 in our in-depth study. Lentivirus-packed plasmids were employed to stably overexpress or knock down DUSP2 in 22RV1. Results of qRT-PCR displayed the transfection efficiency of PCa cells (**Figures 9A, B**). The CCK8 and colony formation assays revealed that 22RV1 cells with DUSP2 upregulation had significantly reduced growth compared to the NC group, and knockdown of MAGI2-AS3 led to the opposite results (**Figures 9C–F**). Additionally, cell migration assay was performed to investigate the regulatory role of DUSP2 in PCa cell migration. We demonstrated that it can inhibit cell migration in the oe-DUSP2 group compared to the control group (**Figure 9G**). However, downregulation of LINC01082 can improve 22RV1 cell migration (**Figure 9H**). These results suggested that DUSP2 is involved in the proliferation and migration of PCa cells.

## 4 Discussion

PCa is among the most prevalent male genitourinary malignancies and remains one of the most common causes of male cancer deaths worldwide (26). Since the utilization of PSA screening, the largest number of patients diagnosed with PCa have demonstrated locoregional disease and therefore benefited from early treatment. While only one-third of newly diagnosed advanced PCa patients in China are spread locally, the rest are diagnosed with invasive PCa with advanced or metastatic symptoms and without surgical opportunities (27). Treatment with androgen deprivation therapy for the distal metastases of hormone-sensitive PCa is the first line, but this carcinoma will eventually develop into CRPC, and the therapeutic drugs are not expected to show long-term remission, thus causing poor prognosis (28). Hence, early diagnosis and treatment are important to improve the prognosis in PCa patients; this prompts us to filter promising biomarkers to clarify the pathogenesis and molecular mechanism of PCa, to find new therapeutic targets and improve patients’ outcome with this disease.

According to reports, a large number of the ceRNA regulatory network have been identified as being involved in the occurrence and development of a variety of human cancers, including lung cancer (29), gastric cancer (30), hepatocarcinoma (31), colorectal cancer (32), ovarian cancer (33), and renal cell carcinoma (34). FOXA1 is a crucial transcription factor functionally involved in the initiation and development of many types of cancers, including PCa. To our knowledge, previous studies about the FOXA1 transcriptional network were mainly focused on protein-coding genes; comprehensive analysis of its regulatory ceRNA network of lncRNAs, however, has not been attempted (35). Accordingly, in this research, we devoted to establishing a FOXA1-related ceRNA triple network in PCa, as well as associating it with prognosis. Contemporaneously, adding evidence has declared that FOXA1 plays a tumorigenesis role and has a prognostic value in PCa; interestingly, we verified similar results through survival analysis, IHC, and copy number variation analysis in this survey.

In this inquiry, firstly, an lncRNA–miRNA–mRNA triple regulatory network comprising 18 lncRNAs, 5 miRNAs, and 44 mRNAs were derived from an *in silico* analysis. Enrichment analysis revealed that the DEmRNAs were primarily concentrated in the “PI3K-Akt signaling pathway”, “receptor ligand activity”, and “urogenital system development”. Following that, a key triple regulatory network, which included five lncRNAs, five miRNAs, and four mRNAs, was identified through hub analysis based on a score >2. Whereafter, the expression analysis and survival analysis of the hub regulatory network were implemented. Furthermore, since the interactions in the ceRNA network mainly take place in the cytoplasm, we also looked at the subcellular localization of the two lncRNAs in the network. After the above bioinformatics analysis, the MAGI2-AS3~hsa-mir-106a/has-mir-204~DUSP2 ceRNA network related to the prognosis of PCa was constructed finally.

By searching for PCAT1 and MAGI2-AS3 in PubMed, we saw that PCAT1 has been explored for roles in PCa or the relationship with PCa. Shang et al. reported an essential role of lncRNA PCAT1 in regulating the PHLPP/FKBP51/IKKα complex to enhance the AKT and NF-κB signaling activity and progression of CRPC (36). Xu et al. confirmed that PCAT1 accelerated PCa cell migration, invasion, proliferation, and suppressed apoptosis by elevating FSCN1 expression mediated *via* miR-145-5p, suggesting a possible therapeutic strategy for PCa patients (37). Consequently, we excluded lncRNA PCAT1 from our ceRNA network since it has been comprehensively studied in the above literature. Meanwhile, according to search results, no studies have been carried out to investigate the role of MAGI2-AS3 in PCa; however, reports on other types of cancer could indirectly explain its roles. For instance, Liu et al. certified that the expression level of MAGI2-AS3 was lessened in breast cancer tissues in contrast to normal adjacent tissues (38). Elevated expression of MAGI2-AS3 restrained the invasive, migratory, and proliferative capabilities, yet facilitated the apoptosis of breast cancer, bladder cancer, and hepatocellular carcinoma cells (38–40). Downregulated MAGI2-AS3 was highly correlated with tumor size, TNM stage, lymph node metastasis, and poor OS. Subsequently, the majority of the studies uncovered that MAGI2-AS3 may regulate their target genes (such as CCDC19, and SMG1) by functioning as a competing endogenous RNA for miRNAs (such as miR-15b-5p and miR-374a/b-5p) in cancers (39, 40). Nonetheless, the mechanism of MAGI2-AS3 remained unclear in PCa. Based on our bioinformatics analysis, we observed that MAGI2-AS3 was consistently downregulated in PCa and predicted that downregulated MAGI2-AS3 may be involved in PCa by functioning as a competing endogenous RNA for has-mir-106a or has-mir-204 to mediate the expression of DUSP2. This hypothesis may lead us to understand that the onco-suppressive role of MAGI2-AS3 extends to PCa.

The dysregulated miRNA has a certain impact on the initiation, development, and prognosis of tumor (41–43). Studies have elucidated that miRNA expression profiles could be used as biomarkers in the early diagnosis, classification, and prognosis of tumors (44). Aberrant expression of miRNAs has been reported in a variety of cancers, including in PCa. In this work, we constructed an lncRNA–miRNA–mRNA network, and the results manifested that the abnormal expression of lncRNAs can cause aberrant expression of five miRNAs, containing has-mir-204, has-mir-206, hsa-mir-106a, has-mir-372, and hsa-mir-93 in PCa, thus leading to mRNA degradation or posttranslational inhibition and therefore regulating the expression of the corresponding protein. After that, through bioinformatics analysis, we eventually included has-mir-106a and has-mir-204 in our network. Hsa-mir-106a was reported to have reduced expression in renal cell carcinoma cells and tissues, and it was proven that the inhibition of hsa-mir-106a expression can enhance cancer cell migration and invasion through interacting with PAK5 (45). Moreover, hsa-mir-106a has previously been certified to be upregulated in many cancer types, including gastric cancer and ovarian cancer (46, 47). Multiple studies have clarified that miR-204-5p can be used as a serum marker for various tumors (48, 49). Notably, an investigation from Daniel et al. has proved that the expression levels of a panel of seven miRNAs, comprising miR-204-5p, in the blood of PCa patients may be used as diagnostic biomarkers for the identification of PCa (50).

Previous studies have reported that immune infiltration can affect the prognosis of patients (51, 52). Therefore, how DUSP2 participates in the tumor microenvironment and influence tumor-infiltrating immune cells in PCa caught our interest. In this study, GSEA for DUSP2 was performed and we found that the tumor immune-related signaling pathways enriched in low expression of DUSP2. Moreover, the infiltration levels of CD4+ T cells and neutrophil cells were increased in the DUSP2 arm-level deletion group in PCa. These differences suggest that the MAGI2-AS3/DUSP2 axis may be closely related to PCa-infiltrated immune cells. Studies have demonstrated that the CTLA-4/B7 and PD-1/PD-L1 axes regulate physiological immune homeostasis, downregulate inflammatory responses, and presumptively facilitate immune evasion of cancer cells (53). Overexpression of PD-1 and CTLA-4 is recognized as a vital suppressor of anti-tumor immunity and is associated with better therapy response (54). In our study, the high expression of DUSP2 was positively correlated with CTLA-4 or/and PD-1 positive; it will be interesting for further studies to explore the relationship between DUSP2 and these ICIs in PCa. Moreover, the associations between DUSP2 and the efficacy of common chemotherapeutics were explored and results indicated that DUSP2 acted as a potential predictor for chemosensitivity.

DUSPs are specialized protein phosphatases that dephosphorylate both tyrosine and serine/threonine residues on the same substrate (55). DUSP2, also called activated cellular phosphatase 1, is a subfamily that acts primarily in the nucleus and predominantly inactivates ERK (56, 57). DUSP2 is mainly expressed in the hematopoietic cells, and it was largely induced by stress responses and regulates cytokine production and inflammation (58). DUSP2 is a transcription factor of the p53 gene in tumor cells, which can regulate cell apoptosis caused by oxidative damage and nutritional stress (59). In some solid tumors, the expression of DUSP2 is downregulated, and it functions as a key downstream regulator of HIF-1-mediated tumor progression and chemoresistance (60). In our exploration, the expression of DUSP2 in clinical samples of PCa was lower than that in BPH tissues, and there was a positive correlation between DUSP2 and MAGI2-AS3 expression in prostate tissues. In contrast to DUSP2 and MAGI2-AS3, has-mir-106a and has-mir-204 expression were upregulated in PCa compared to BPH tissues. On the side, we performed a molecular validation *via* functional experiments, and the results indicated that DUSP2 could regulate the proliferation and migration of PCa cells, which complement the mechanism of MAGI2-AS3 in the pathogenesis and development of PCa.

Although we have constructed a ceRNA-based molecular marker for MAGI2-AS3/DUSP2, there are still some limitations that should be noted. Firstly, the small number of PCa or BPH tissue samples would impact our findings’ credibility. Secondly, further experiments are needed to determine the binding affinity between lncRNAs, miRNAs, and mRNAs obtained from databases. Last but not least, the relationship between DUSP2 and ICIs needs to be elucidated in further experiments to explore the PCa mechanism.

## 5 Conclusions

In this investigation, a ceRNA network (MAGI2-AS3~hsa-mir-106a/hsa-mir-204~DUSP2) related to PCa prognosis was created to further understand the correlation of ceRNA, and the prognostic model is useful for exploring the pathogenesis of PCa. Otherwise, we identified that the lncRNA MAGI2-AS3 can be a novel vital factor in diagnosing PCa and determining the prognosis of PCa patients. In addition, DUSP2 was important for immune cell infiltration and chemosensitivity. Our study provides novel insights into immunological biomarkers and improves our understanding of FOXA1-related ceRNA in PCa.

## Data availability statement

The original contributions presented in the study are included in the article/**Supplementary Material**. Further inquiries can be directed to the corresponding authors.

## Ethics statement

The studies involving human participants were reviewed and approved by Ethics Committee of the First Affiliated Hospital of Chongqing Medical University. The patients/participants provided their written informed consent to participate in this study. Written informed consent was obtained from the individual(s) for the publication of any potentially identifiable images or data included in this article.

## Author contributions

GY, XC, JL, and YZ designed the study. GY and YZ wrote the manuscript and analyzed data. GY, XC, and YZ performed the bioinformatics analysis. LP, YG, YT, LW, and YW collected the samples and verified the gene expression level. ML conducted the HE and IHC. JL and YZ wrote, reviewed, and edited the paper. ZQ and XW supervised and provided research funding for the learning. All authors contributed to the article and approved the submitted version.
