# Supplementary material for: Comprehensive analysis of the FOXA1-related ceRNA network and identification of the MAGI2-AS3/DUSP2 axis as a prognostic biomarker in prostate cancer
Source: Front Oncol. 2023 Mar 14;13:1048521. doi: 10.3389/fonc.2023.1048521 (PMC10043306; doi:10.3389/fonc.2023.1048521)
Supplement: Supplementary file 1 [file DataSheet1.docx]

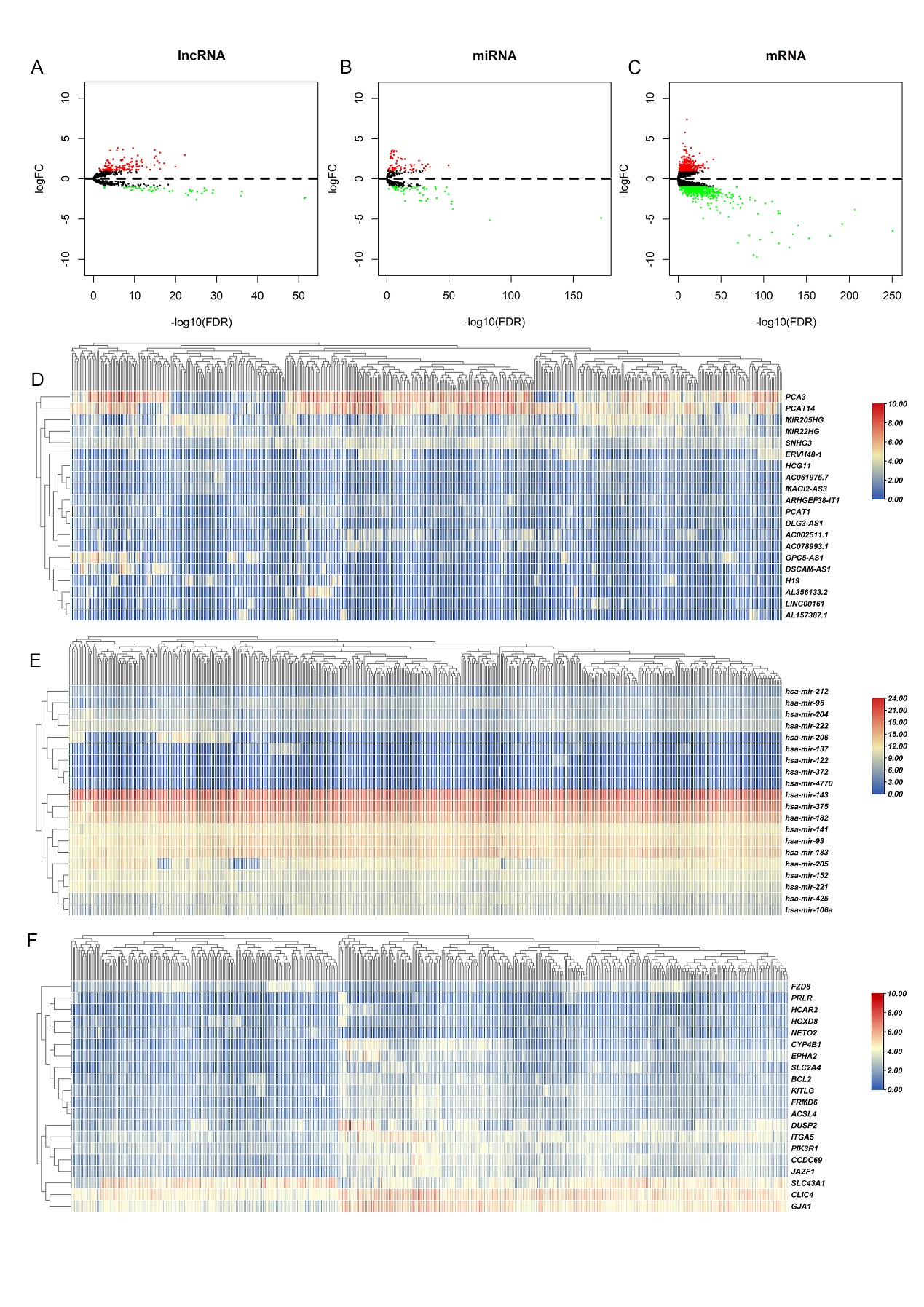
**Supplementary Figure 1. Volcano plots and heatmap plots of DElncRNAs, DEmiRNAs, and DEmRNAs between TCGA-PRAD tumor and normal samples.** Red represents upregulated genes and blue indicates downregulated genes. (A–C) The volcano plots describe (A) 178DElncRNAs (|log2fold change| > 1 and adjusted *p* value < 0.05), (B) 123DEmiRNAs (|log2fold change| > 1 and adjusted *p* value <0.05), and (C) 1059DEmRNAs (|log2fold change| > 1 and adjusted *p* value < 0.05). (D–F) The horizontal axis of the heatmap indicates the samples, and the vertical axis of the heatmap indicates 15 significant DEGs


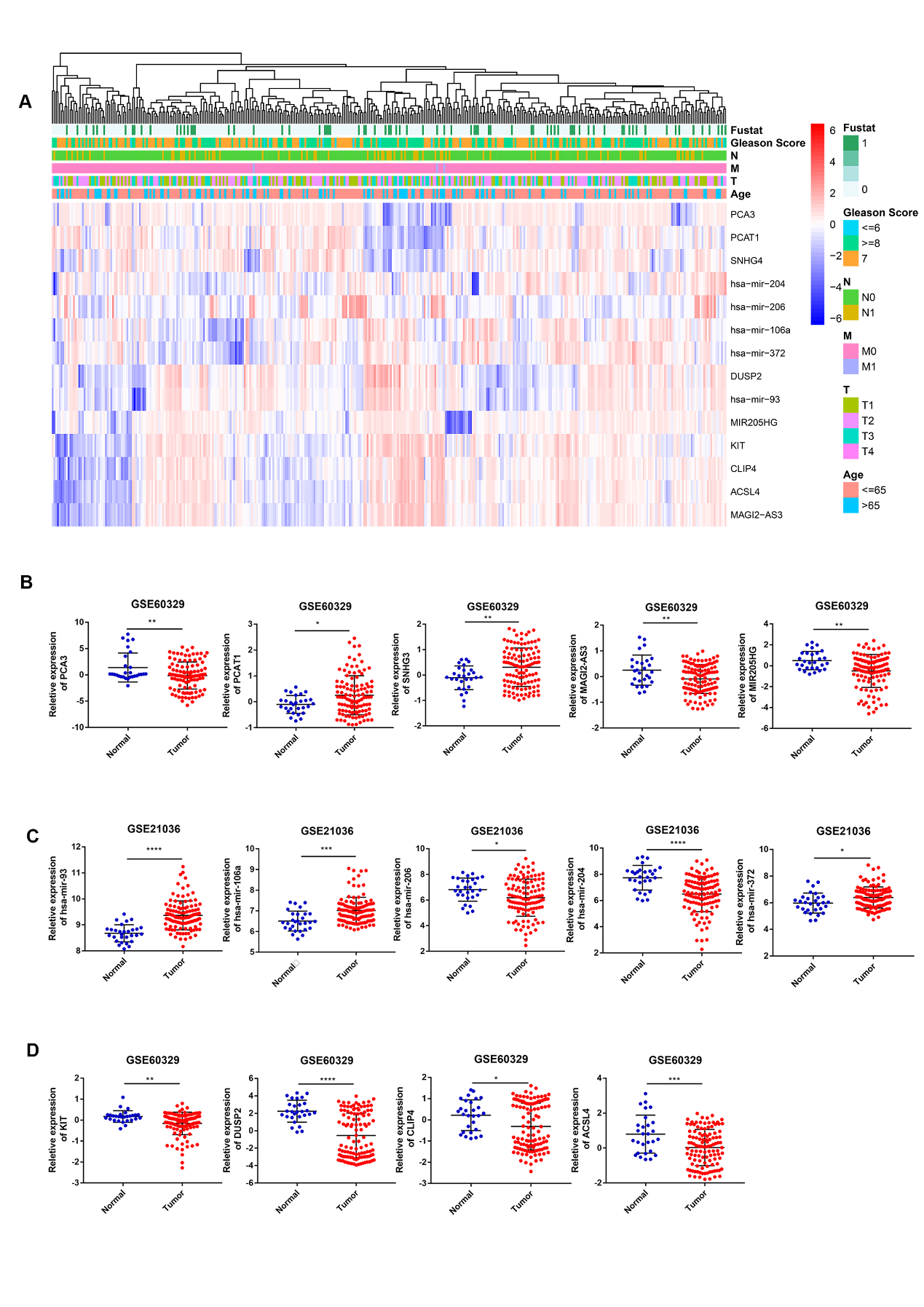
**Supplementary Figure 2. Expression analysis for the hub genes by GEO dataset.** (A) The associated between hub genes and the clinical characteristics of PCa visualized by heatmap. (B-D) The expression patterns of hub genes in PCa and normal prostate tissues. (B) five hub-DElncRNAs, (C) five hub-DEmiRNAs, and (D) four hub- DEmRNAs.
