# Supplementary material for: Comprehensive analysis of the FOXA1-related ceRNA network and identification of the MAGI2-AS3/DUSP2 axis as a prognostic biomarker in prostate cancer
Source: Front Oncol. 2023 Mar 14;13:1048521. doi: 10.3389/fonc.2023.1048521 (PMC10043306; doi:10.3389/fonc.2023.1048521)
Supplement: Supplementary file 2 [file Table_1.docx]

Supplementary table 1: Immunohistochemistry of FOXA1 in Human Protein Atlas database.

| No. | Tissue type | Patient ID | Age | Staining |
| --- | --- | --- | --- | --- |
| 1 | tumor | 3454 | 57 | high |
| 2 | tumor | 3958 | 60 | high |
| 3 | tumor | 4327 | 68 | high |
| 4 | normal | 3497 | 37 | medium |
| 5 | normal | 3316 | 55 | medium |
| 6 | normal | 1984 | 61 | medium |

Supplementary table 2: Top 10 significantly enriched pathways about GO and KEGG derived from the DEmRNAs.

| Ontology | ID | Description | P value | P.adjust | Q value |
| --- | --- | --- | --- | --- | --- |
| BP | GO:0003012 | muscle system process | 6.77054E-20 | 1.76034E-16 | 1.43535E-16 |
| BP | GO:0006936 | muscle contraction | 1.98353E-21 | 1.03143E-17 | 8.41015E-18 |
| BP | GO:0007517 | Muscle organ development | 9.73229E-10 | 1.01216E-06 | 8.25298E-07 |
| BP | GO:0001655 | urogenital system development | 4.25749E-10 | 5.53474E-07 | 4.51294E-07 |
| BP | GO:0048732 | gland development | 4.20245E-06 | 0.000335345 | 0.000273436 |
| BP | GO:1903522 | regulation of blood circulation | 1.41341E-10 | 2.44991E-07 | 1.99762E-07 |
| BP | GO:0022898 | regulation of transmembrane transporter activity | 4.02422E-05 | 0.001851852 | 0.001509972 |
| BP | GO:0034764 | positive regulation of transmembrane transport | 3.93775E-05 | 0.001828243 | 0.001490721 |
| BP | GO:1901379 | regulation of potassium ion transmembrane transport | 4.26679E-05 | 0.001912698 | 0.001559585 |
| BP | GO:0086065 | cell communication involved in cardiac conduction | 5.21547E-07 | 8.47514E-05 | 6.9105E-05 |
| CC | GO:0062023 | collagen-containing extracellular matrix | 3.57467E-14 | 9.16904E-12 | 7.52563E-12 |
| CC | GO:0005924 | cell-substrate adherens junction | 3.08446E-08 | 1.43848E-06 | 1.18066E-06 |
| CC | GO:0005925 | focal adhesion | 2.44472E-08 | 1.25414E-06 | 1.02936E-06 |
| CC | GO:0043292 | contractile fiber | 8.90803E-14 | 1.52327E-11 | 1.25025E-11 |
| CC | GO:0044449 | contractile fiber part | 2.42695E-13 | 3.11256E-11 | 2.55468E-11 |
| CC | GO:0030016 | myofibril | 7.3292E-12 | 7.51975E-10 | 6.17195E-10 |
| CC | GO:0030017 | sarcomere | 1.32917E-10 | 9.74091E-09 | 7.995E-09 |
| CC | GO:0009898 | cytoplasmic side of plasma membrane | 8.3186E-05 | 0.001524087 | 0.001250918 |
| CC | GO:0031234 | extrinsic component of cytoplasmic side of plasma membrane | 4.6527E-07 | 1.49177E-05 | 1.2244E-05 |
| CC | GO:0016010 | dystrophin-associated glycoprotein complex | 0.000234264 | 0.003081475 | 0.002529168 |
| MF | GO:0048018 | receptor ligand activity | 0.000297242 | 0.007013305 | 0.005953297 |
| MF | GO:0005539 | glycosaminoglycan binding | 5.00039E-12 | 2.18267E-09 | 1.85277E-09 |
| MF | GO:0008201 | heparin binding | 5.57503E-11 | 1.62234E-08 | 1.37713E-08 |
| MF | GO:0005262 | calcium channel activity | 0.000254757 | 0.006354376 | 0.00539396 |
| MF | GO:0005200 | structural constituent of cytoskeleton | 0.000292264 | 0.007013305 | 0.005953297 |
| MF | GO:0019199 | transmembrane receptor protein kinase activity | 0.000856725 | 0.016620463 | 0.014108405 |
| MF | GO:0004714 | transmembrane receptor protein tyrosine kinase | 0.000360082 | 0.008272412 | 0.007022099 |
| MF | GO:0048306 | calcium-dependent protein binding | 0.001250254 | 0.023110965 | 0.019617917 |
| MF | GO:0098632 | cell-cell adhesion mediator activity | 0.00024075 | 0.006181609 | 0.005247305 |
| MF | GO:0098641 | cadherin binding involved in cell-cell adhesion | 0.002544954 | 0.037358225 | 0.031711811 |
| KEGG | hsa04151 | PI3K-Akt signaling pathway | 0.000198081 | 0.004433966 | 0.003576685 |
| KEGG | hsa04510 | Focal adhesion | 4.3811E-10 | 1.2749E-07 | 1.02841E-07 |
| KEGG | hsa04810 | Regulation of actin cytoskeleton | 0.001280673 | 0.023292247 | 0.018788826 |
| KEGG | hsa04020 | Calcium signaling pathway | 0.000951674 | 0.018462481 | 0.014892868 |
| KEGG | hsa04310 | Wnt signaling pathway | 0.001672605 | 0.028631054 | 0.023095406 |
| KEGG | hsa04921 | Oxytocin signaling pathway | 0.0026166 | 0.034610478 | 0.027918744 |
| KEGG | hsa04915 | Estrogen signaling pathway | 0.00194239 | 0.029749229 | 0.023997388 |
| KEGG | hsa05412 | Arrhythmogenic right ventricular cardiomyopathy | 4.72012E-06 | 0.000228926 | 0.000184664 |
| KEGG | hsa04974 | Protein digestion and absorption | 0.001883059 | 0.029749229 | 0.023997388 |
| KEGG | hsa04929 | GnRH secretion | 0.000820554 | 0.017055808 | 0.013758167 |

Supplementary table 3: The detail of predict the binding site of RNA-RNA interaction.

| Target | Position | Query | Position | Energy(kcal/mol) |
| --- | --- | --- | --- | --- |
| MAGI2-AS3 | 339-357 | Has-mir-106a | 47-33 | -8.04161 |
| MAGI2-AS3 | 448-460 | Has-mir-204 | 19-8 | -9.97361 |
| DUSP2 | 65-76 | Hsa-mir-106a | 48-35 | -4.65723 |
| DUSP2 | 69-86 | Has-mir-204 | 17-1 | -12.2355 |

Supplementary table 4: Association between DUSP2 and clinical features of prostate cancer patients.

| **Variables** | Cases | DUSP2 expression | | **P‑value** |
| --- | --- | --- | --- | --- |
|  |  | Low(n=177) | High(n=177) |  |
| Age(year) | | |  |  |
| <60 | 136 | 76(42.9%) | 60(33.9%) | 0.080 |
| ≥60 | 218 | 101(57.1%) | 117(66.1%) |  |
| Postoperrative PSA (ng/ml) | | | |  |
| <10 | 341 | 174(98.3%) | 167(94.4%) | 0.086 |
| ≥10 | 13 | 3(1.7%) | 10(5.6%) |  |
| TNM stage | | |  |  |
| I/II | 123 | 59(33.3%) | 64(35.4%) | 0.678 |
| III/ IV | 231 | 118(66.7%) | 113(64.6%) |  |
| Gleason score | | |  |  |
| ≤7 | 124 | 52(39.4%) | 72(40.7%) | 0.026* |
| ≥7 | 230 | 125(70.6%) | 105(59.3%) |  |
| Lymph-node Metastases | | | |  |
| Negative | 288 | 144(81.4%) | 144(81.4%) | 1.000 |
| Positive | 66 | 33(18.6%) | 33(18.6%) |  |
| Distant Metastases | | |  |  |
| Negative | 342 | 167(94.4%) | 175(98.9%) | 0.035* |
| Positive | 12 | 10(5.6%) | 2(1.1%) |  |

* Statistically significant (*P*＜0.05).
